# Customized Hybrid Bluegrass Appliance: An Innovative Technique

**DOI:** 10.5005/jp-journals-10005-1500

**Published:** 2018-04-01

**Authors:** Ziauddin Mohammad, Apeksha Bagalkotkar, Ashank Mishra, Gopi Veerala

**Affiliations:** 1 Associate Professor, Department of Pedodontics and Preventive Dentistry, Sri Sai College of Dental Surgery, Hyderabad, Telangana, India; 2 Associate Professor, Department of Pedodontics and Preventive Dentistry, Sri Sai College of Dental Surgery, Hyderabad, Telangana, India; 3 Associate Professor, Department of Periodontics, Sri Sai College of Dental Surgery Hyderabad, Telangana, India; 4 Consultant, Department of Orthodontist, Sree Anjaneya Institute of Dental Sciences, Modakkallur, Kerala, India

**Keywords:** Bluegrass, Esthetics, Functional space maintainer, Natural tooth pontic, Oral habits.

## Abstract

Oral habits in the form of thumb sucking and tongue thrusting are commonly learned patterns of behavior seen in preschool children and they are associated with anxiety, fear, hunger, oral pressure, and sleep. Chronic practice can cause dentoalveolar, perioral problems, and atypical root resorption (ARR) of anterior primary teeth. The ARR is provoked by the thumb sucking habit, and leads to early loss of anterior primary teeth. The early loss of anterior tooth may result in speech and masticatory problems, and psychological disturbance to the child. Hence, pediatric dentists play a crucial role in giving necessary information to parents and guardians. Starting from counseling to appliance therapy, various treatment modalities have been reported in the literature. One of them is bluegrass appliance; it is a nonpunitive habit reminder therapy. The present case report describes a customized hybrid bluegrass appliance designed to eliminate thumb sucking and tongue thrusting habit, and to perform as an esthetic functional space maintainer.

**How to cite this article:** Mohammad Z, Bagalkotkar A, Mishra A, Veerala G. Customized Hybrid Bluegrass Appliance: An Innovative Technique. Int J Clin Pediatr Dent 2018;11(2):141-145.

## INTRODUCTION

A habit is a repetitive action that is being done involuntarily. Repetitive behaviors are common in the infantile period and most of them are started and finished instinctively. The thumb sucking habit is normal at the age of 2 to 3 years of life; beyond this time, it may cause adverse effects on dentition and dental bearing areas.^1^ The severity of the habit depends on the duration, intensity, and frequency. The prolonged thumb sucking habit could lead to compensate tongue thrusting habit.^2^ Taylor and Peterson^3^ reported that digit sucking appears to contribute to the development of ARR of the maxillary primary central incisors, and this finding was confirmed by Rubel.^4^ The ARR of the maxillary primary incisor is characterized by superficial root resorption along the lateral and apical aspects of the roots of these teeth, and extraction of these teeth is inevitable in extreme conditions.^5^ Early extractions of these teeth may lead to a psychological trauma to the child and parents. Early intervention of thumb sucking and tongue thrusting habit could eliminate the adverse events that occur on the dentition, dental bearing, and perioral musculatures. This paper presents a case report of a child with thumb sucking, compensatory tongue thrusting habit, along with ARR and its management.

## CASE REPORT

A 4-year-old female child accompanied by her mother reported to a private clinic with a chief complaint of thumb sucking habit. Detailed history revealed from her mother indicated that the child used to suck her thumb when she felt bored and while sleeping. Her mother tried to stop the habit by applying a bitter neem oil substance over her thumb, which was unsuccessful.

**Figs 1A to C:**
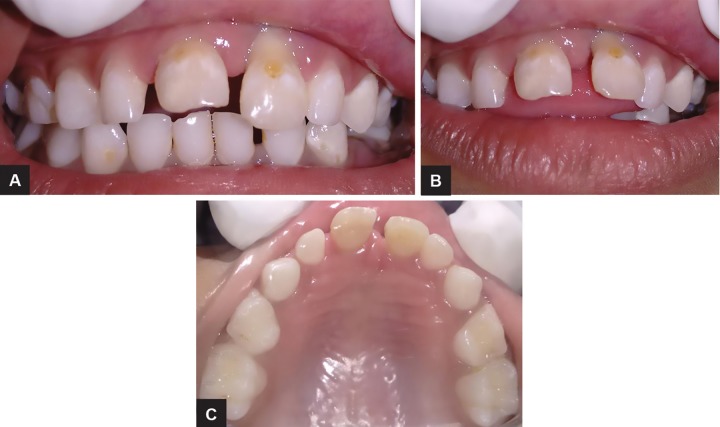
Intraoral photographs

**Fig. 2:**
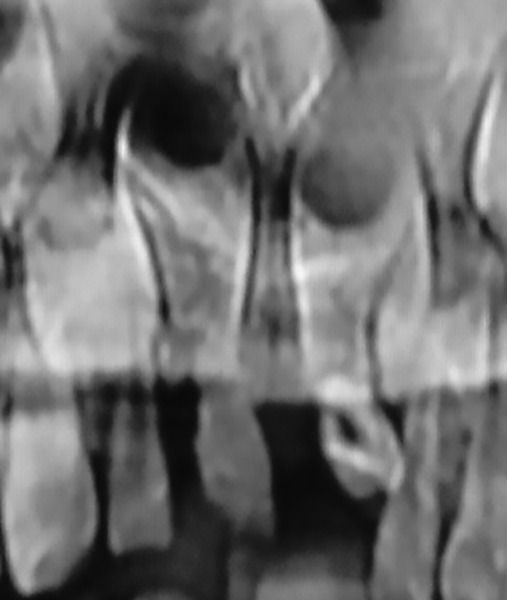
Intraoral periapical radiograph irt 51, 61

The extraoral examination of the patient showed good facial symmetry and convex profile. The intraoral examination revealed anterior open bite, average-sized tongue, and proclination of maxillary anterior teeth; grade III mobility was seen with maxillary central incisors, and while swallowing, the tongue was placed in between maxillary and mandibular anterior teeth (tongue thrusting habit) (Fig. 1). The intraoral periapical radiograph revealed root resorption along the lateral and apical aspects of maxillary central incisors (Fig. 2). It was diagnosed based on the clinical and radiographic finding, ARR accompanied with thumb sucking, and compensated tongue thrusting habit.

The detailed treatment plan was formulated and explained to the mother and her consent was obtained. Local anesthesia was administered prior to the extraction of maxillary central incisors (LIGNOX 2% A, adrenaline, Lignocaine 1: 80000, Lic No: 557, B. No: LAK2K42, Indoco remedies Ltd). The extracted teeth were cleaned and preserved in saline, and the patient was scheduled after a week for further treatment. Treatment of thumb sucking and tongue thrusting was initiated on the second appointment by counseling the parent and the child regarding the adverse effect of the habits on the developing dentition. Based on the parental esthetic concern, we planned a habit reminder therapy using a customized bluegrass appliance with natural tooth pontics as a functional esthetic space maintainer. This modification justified both parental esthetic concern and habit reminder therapy.

## APPLIANCE DESIGN

**Figs 3A to C:**
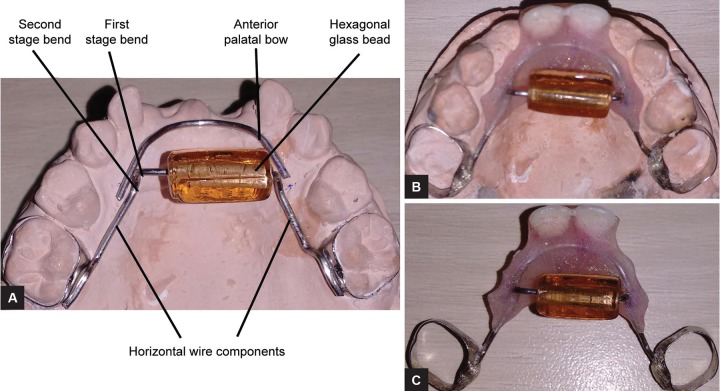
Customized hybrid bluegrass appliance design and fabrication

The orthodontic bands (3M Unitek, USA) were placed on 55, 65 and an alginate impression was made, bands were stabilized in alginate impression, and a working cast was prepared. A modified bluegrass appliance was fabricated by adapting 0.9 mm stainless steel wire over the palate extending from the second primary molar on either side. A rectangular hexagonal glass bead was inserted into the stainless steel wire and a first-stage downward right angle bends were given on either side of the bead; the second-stage 45° bends were given 5 mm from the first-stage bend on either side, and no contact was established by bead with palatal tissues. The wire was soldered to the molar bands. Another (palatal bow) 0.9 mm stainless steel wire was adapted to the premaxillary area on either side of the bluegrass appliance; the distal extensions of wire component were soldered to the second-stage bluegrass wire (Fig. 3); the benefit of this added modification was to use it as a functional space maintainer.

## NATURAL TOOTH PONTICS PREPARATIONS

**Figs 4A to D:**
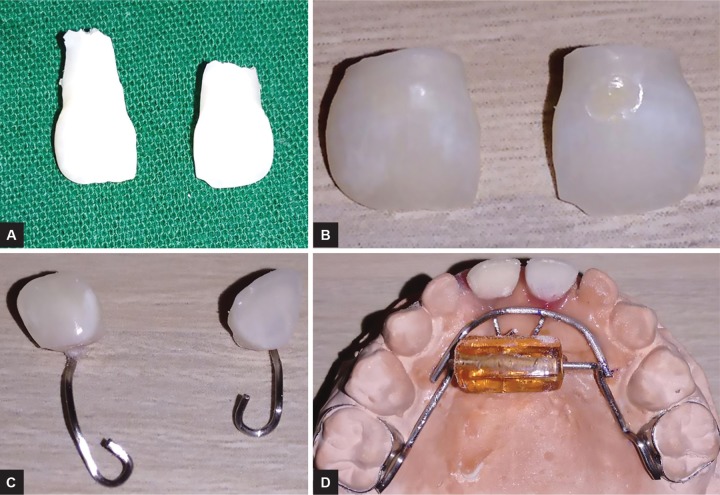
(A) Extracted 51, 61. (B) Prepared natural tooth pontics. (C) Stainless steel wire engaged in the pulp chamber of 51, 61. (D) Pontics stabilized in acrylic component

**Figs 5A to F:**
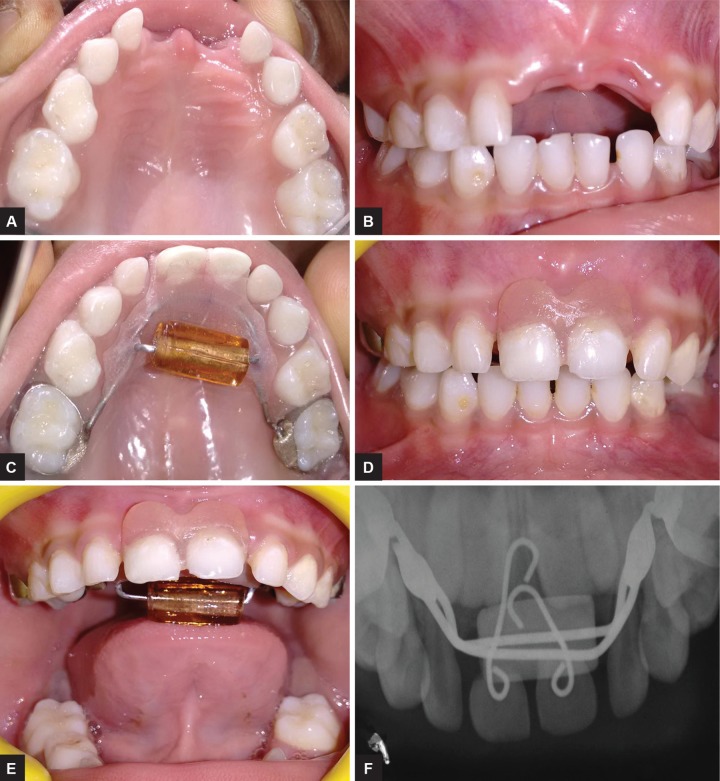
Customized hybrid bluegrass appliance cemented on 55, 65 and anterior occlusal radiograph

The access cavity preparation was done in 51, 61, and the complete necrotic pulp was removed. Roots of the central incisors were resected with a straight fissure bur and discarded. A 0.6 mm stainless steel wire was engaged in the pulp chamber of 51, 61 as shown in the picture, and the other end of the wire extended till the 0.9 mm wire and embedded in the acrylic (Fig. 4). To hold the pontic in place, for better stability, the acrylic component was used as a base for the prepared natural tooth pontics. By the third appointment, the appliance was cemented on 55 and 65 using type I glass ionomer cement (GC I, GC Corporation, Tokyo, Japan) to the 55, 65 (Fig. 5). The patient was instructed to roll the bead with her tongue, whenever she felt like sucking her thumb and oral hygiene instructions were given. Recall check-ups were scheduled initially every week for the 1st month, later for every 1 month. The appliance was found to be functioning satisfactorily inside the oral cavity for the last 4 months.

## DISCUSSION

Thumb sucking usually involves placing the thumb into the mouth and rhythmically repeating sucking contact for a prolonged duration and is considered to be relaxing and beneficial for the person.^6^ Tongue thrusting habit is defined as a human behavioral pattern in which the tongue protrudes through the anterior teeth during the swallowing pattern, speech, and rest.^7^ Both of these habits are considered to be normal up to 3 to 4 years of age.^8^ But it can lead to deleterious effects in the oral cavity if these habits persist beyond. The severity of deleterious effects on the oral cavity depends on the habit, duration, intensity, and frequency.^9^

So, the intervention of habits is necessary when it is seen earlier. Taylor and Peterson^3^ agreed that an association seems to exist between the presence of a digit sucking and ARR on primary maxillary central incisors as seen in the radiograph, and this may be the result of long duration and more intensity of digit habit. Furthermore, it is also possible that exfoliation-related root resorption might be hastened by the digit sucking habit. Holan et al^9^ suggested that, the association of dental trauma with ARR in primary incisors, but not with digit sucking.

In the present case, the intensity of habit was severe, the maxillary central incisors were grade III mobility, and indicated for extraction. The mother was concerned particularly about esthetics, so we modified the bluegrass appliance, as it was used both for habit breaking and as a functional space maintainer.

The customized hybrid bluegrass appliance is a compound appliance with multiple advantages. This single appliance can be used to treat thumb sucking as well as tongue thrusting and replacing the extracted teeth. The bluegrass appliance acts as a habit breaker, where the hexagonal bead serves as a training device in the oral cavity with tongue thrusting, which eventually guides the position of the tongue. In the case of thumb sucking, the hexagonal bead can serve as a reminder.

Another advantage of this appliance is as a functional space maintainer. We incorporated a functional space maintainer to the bluegrass appliance using the patient’s extracted tooth. When the extracted tooth is used, it has an added advantage of preservation of natural tooth form, contour, and translucency, and thereby renders psychological benefit to the child. One more advantage of this appliance is that, once the purpose of bluegrass appliance is achieved, then we can remove the glass bead, keeping the functional space maintainer in the oral cavity until its need.

## CONCLUSION

The customized hybrid bluegrass appliance is a multipurpose appliance and it can be effectively used to correct thumb sucking as well as tongue thrusting habits. Adding functional esthetic space maintainer to the bluegrass appliance gives an additional advantage to address the esthetic demands of the child and the parent.
